# Prophylactic surfactant therapy in the era of less invasive surfactant delivery

**DOI:** 10.1038/s41372-025-02420-z

**Published:** 2025-09-23

**Authors:** Dinushan C. Kaluarachchi, Anup Katheria, Patrick J. Peebles, Scott O. Guthrie, Venkatakrishna Kakkilaya, Peter A. Dargaville

**Affiliations:** 1 Department of Pediatrics, University of Wisconsin – Madison, Madison, WI USA; 2 Neonatal Research Institute, Sharp Mary Birch Hospital for Women & Newborns, San Diego, CA USA; 3 Division of Neonatology, Department of Pediatrics, Vanderbilt University School of Medicine, Nashville, TN USA; 4 Division of Neonatology, Department of Pediatrics, Jackson-Madison County General Hospital, Jackson, TN USA; 5 University of Texas Southwestern Medical Center, Dallas, TX USA; 6 Menzies Institute for Medical Research, University of Tasmania, Hobart, TAS Australia; 7 Department of Paediatrics, Royal Hobart Hospital, Hobart, TAS Australia

**Keywords:** Respiratory tract diseases, Paediatrics

## Abstract

Pulmonary surfactant is a lifesaving treatment for preterm infants with respiratory distress syndrome (RDS). With the advent of less-invasive surfactant delivery techniques that minimize or eliminate exposure to invasive mechanical ventilation, the question of prophylactic surfactant administration has reemerged. This review discusses the evidence for less-invasive surfactant therapy applied prophylactically. In summary, prophylactic surfactant administration by pharyngeal deposition or brief intubation are not recommended. Surfactant delivery via supraglottic airway or by aerosolization have not been investigated as prophylaxis for RDS. Recent evidence suggests that prophylactic surfactant administration via thin catheter is safe and effective. Several ongoing randomized clinical trials are further evaluating the efficacy of prophylactic surfactant administration via thin catheter. Prophylactic surfactant administration via supraglottic airway devices and aerosolization should be evaluated in future studies.

## Introduction

Exogenous surfactant delivered intratracheally is a highly effective treatment for preterm infants with respiratory distress syndrome (RDS), reducing mortality and the risk of air leaks [1, 2]. Surfactant administration soon after birth via an endotracheal tube (ETT) was once considered a standard practice for preterm infants [3]. However, delivery room intubation rates have decreased significantly following clinical trials demonstrating no advantage of this practice compared with noninvasive respiratory support for infants <30 weeks’ gestation [4]. Since the shift away from routine intubation, the optimal timing and thresholds for surfactant administration remain controversial [5, 6]. With the widespread adoption of continuous positive airway pressure (CPAP) support in early life, new methods for less-invasive surfactant delivery have emerged [7]. Ongoing and existing trials are examining whether these techniques can be applied prophylactically in selected preterm infant populations. This review evaluates the evidence for prophylactic surfactant therapy combined with various less-invasive administration techniques.

## Prophylactic surfactant prior to routine use of antenatal steroids and CPAP

Surfactant replacement therapy was first investigated in human infants in the 1960s using aerosolized products with poor spreading properties [8, 9]. Following extensive research characterizing the biophysical and biochemical nature of surfactant and its deficiency in RDS, numerous controlled clinical trials of exogenous surfactant therapy were completed in the 1980s [10]. Both rescue surfactant for infants admitted to the neonatal intensive care unit (NICU) and prophylactic surfactant therapy in the delivery room were investigated to reduce RDS severity and mortality. Compared with untreated controls, these trials consistently demonstrated benefits, particularly reduced mortality and risk of air leak syndromes [1].

Some early trials compared prophylactic surfactant administration in the delivery room with early rescue therapy. These studies showed promising results, with nonselective delivery room administration reducing the risk of pneumothorax, pulmonary interstitial emphysema, mortality, and bronchopulmonary dysplasia (BPD) or death compared with selective rescue therapy for infants with RDS [10]. However, these trials were conducted before the routine use of antenatal steroids. Additionally, most participants assigned to early rescue therapy were intubated in the delivery room and mechanically ventilated before receiving surfactant.

## Prophylactic surfactant in the era of routine antenatal steroids and CPAP

As the benefits of CPAP became evident, clinical trials in the early 2000s compared routine delivery room intubation with first-intention CPAP in preterm infants <30 weeks’ gestation [11–14]. These trials, conducted when maternal steroid use was more common, found that most (approximately 90%) of infants randomized to delivery room intubation received prophylactic surfactant, whereas about 50% of those in the CPAP arm required surfactant after intubation usually due to evolving RDS. A meta-analysis showed that first-intention CPAP with selective surfactant administration reduced the risk of BPD or death compared with routine intubation [15].

These findings have significantly influenced early respiratory management in preterm infants. Initiating CPAP immediately after birth with selective surfactant administration is now considered an alternative to routine intubation with prophylactic or early surfactant. Emphasis is placed on avoiding mechanical ventilation to prevent lung injury from volutrauma, barotrauma, and oxygen toxicity. CPAP support conserves endogenous surfactant which is turned over more rapidly with mechanical ventilation. Moreover, surfactant deficiency in preterm infants is not an all-or-nothing phenomenon; many infants have sufficient surfactant synthetic capability and will benefit from CPAP without the need of exogenous surfactant administration.

## Disadvantages of routine stabilization on CPAP and selective surfactant

Despite modest benefits in studied populations, first-intention CPAP has limitations. A significant proportion of extremely preterm infants experience CPAP failure, requiring surfactant and mechanical ventilation. In randomized clinical trials, 15–67% of infants in the CPAP group developed CPAP failure and received surfactant [11–14]. In a population-based study in Australia and New Zealand, Dargaville et al. reported that 43% of infants born at 25–28 weeks experienced CPAP failure within 72 h, requiring intubation, often before 24 h [16]. Infants with CPAP failure faced increased risks of pneumothorax, prolonged respiratory support, chronic lung disease, and extended hospital stays [16].

## Prophylactic surfactant with INSURE

Early prophylactic surfactant trials typically involved surfactant administration followed by mechanical ventilation. The INSURE (INtubation, SURfactant, Extubation) approach [17, 18] is the most common method to deliver surfactant and has been explored in several clinical trials as a way to administer prophylactic surfactant [13, 14, 19]. A meta-analysis found no evidence that early INSURE or nasal CPAP alone was superior [20]. Relative risk estimates favored early INSURE, with a 12% reduction in chronic lung disease or death, a 14% reduction in chronic lung disease, and a 50% reduction in air leaks, though these findings were not statistically significant. The authors suggested these results may still be clinically important [20].

A limitation of INSURE is that rapid extubation may not always be achievable, exposing infants to positive pressure ventilation. In published studies, it is generally unclear how quickly the endotracheal tube was removed in subjects who received surfactant via INSURE. Even in a clinical trial setting, 10–17% of infants were unable to be extubated as intended [13, 14]. This percentage is likely much higher in clinical practice. As shown in preclinical studies, even brief exposure to endotracheal mechanical ventilation can cause lung injury and edema [21].

## Surfactant administration via thin catheter

Surfactant administration via thin catheter, also known as less-invasive surfactant administration (LISA) or minimally invasive surfactant therapy (MIST), delivers surfactant to spontaneously breathing, non-intubated infants while avoiding intratracheal positive pressure ventilation.⁷ We will refer to this technique as LISA for the rest of this manuscript. LISA maintains noninvasive respiratory support and, in most cases, avoids positive pressure ventilation. Its safety and efficacy have been demonstrated in multiple clinical trials and extensive experience within the German Neonatal Network (GNN) [22–26].

## Other less-invasive methods of surfactant administration

Other noninvasive surfactant administration techniques include pharyngeal surfactant instillation, which a recent study found ineffective. Infants receiving oropharyngeal surfactant at birth were more likely to develop pneumothorax [27]. Aerosolized surfactant has shown promise in more mature preterm infants but lacks efficacy in extremely preterm infants [28–30]. Cummings et al. reported that aerosolized calfactant for mild to moderate respiratory distress reduced the need for intubation and liquid surfactant by nearly 50% [28].

Surfactant administration via supraglottic airway devices is promising for more mature preterm infants but has not yet been studied in extremely preterm infants due to the lack of appropriately sized devices [31, 32]. However, the U.S. Food and Drug Administration recently cleared a novel supraglottic airway device for neonates <1000 g (https://www.accessdata.fda.gov/scripts/cdrh/cfdocs/cfRL/rl.cfm?lid=933470&lpcd=CAE). Given the minimally invasive nature of these methods compared with laryngoscopy, further research and development are needed to benefit extremely preterm infants.

## LISA vs. INSURE

LISA and INSURE are the most studied less-invasive surfactant administration techniques for preterm infants. A meta-analysis comparing these methods found LISA associated with a reduced risk of death or BPD and less intubation within 72 h [26]. These results have led major clinical practice guidelines to recommend LISA as the preferred method [33, 34].

De Luca et al. highlighted methodological flaws in trials comparing LISA to INSURE, arguing that none compared LISA with optimally performed INSURE, defined as short-duration (10–15 min), gentle ventilation with controlled inspiratory pressure and extubation to adequate CPAP or noninvasive positive pressure ventilation [35]. De Luca et al. argues that CPAP transmission is not assured during the LISA procedure, and hence dispersal of surfactant from the trachea may not be optimized.

## Prophylactic LISA

LISA offers a method to administer surfactant with minimal or no exposure to positive pressure ventilation. Limited data exist on prophylactic LISA in the delivery room. Within GNN NICUs, surfactant delivery via LISA soon after birth is common, bundled with early caffeine administration and practices facilitating extrauterine transition, including delayed cord clamping and higher CPAP levels [25]. Among 6542 infants born at 22–26 weeks, 2534 (39%) received LISA, with 83% of procedures performed in the delivery room as quasi-prophylactic, without a set FiO_2_ threshold. Nearly half (46%) of LISA recipients required mechanical ventilation within 72 h, reflecting their low gestational age. Compared with infants not receiving LISA (88% of whom received surfactant via ETT), those receiving LISA had reduced risks of all-cause death, BPD, and BPD or death [25].

To date, the CALI trial is the only published clinical trial to investigate early routine LISA compared to a control group managed expectantly with first intention CPAP [36]. Caffeine administration had to be completed prior to LISA according to the trial protocol. Among 92 infants born at 24–29 weeks receiving early routine LISA, 21 (23%) developed CPAP failure within 72 h and required intubation, compared with 47 of 88 (53%) in the CPAP group. The BPD rate was lower in the LISA arm (26% vs. 39%, P = 0.04). Although randomized early (mean age 7 min), surfactant delivery occurred at a median of 1.5 h due to the caffeine requirement, so the study does not fully examine prophylactic administration as in early surfactant studies [36].

## Early routine vs. early targeted LISA

Prophylactic or early routine surfactant via LISA is a promising strategy to administer surfactant early, potentially preventing lung injury. Initial studies show reduced CPAP failure and BPD incidence. This approach requires evaluation in larger randomized clinical trials, several of which are underway (Table 1) [37] (https://clinicaltrials.gov/study/NCT04715373), (https://clinicaltrials.gov/study/NCT06007547). Given that some infants can be managed with CPAP alone, whether LISA should be routine or targeted remains unclear. Future studies should identify infants at highest risk of RDS based on perinatal variables, as they are most likely to benefit from early surfactant therapy.

**Table 1:** Ongoing studies of prophylactic surfactant administration via thin catheter.

Study name, reference	Patient population	Active treatment arm	Comparator group	Primary outcome	Planned sample size
Pro.LISA [35]	25 0/7 to 28 6/7 weeks’ gestation, supported with CPAP in first hour, FiO_2_ < 0.30	Prophylactic surfactant 100–200 mg/kg via thin catheter	Continuation of CPAP, surfactant to be given via thin catheter if FiO_2_ ≥ 0.30 or if respiratory distress observed by physician	FEV_1_ at 5 years	698 infants
DR LISA [36] (NCT04715373)	22 0/7 to 25 6/7 weeks’ gestation	Prophylactic surfactant 200 mg/kg via thin catheter	Continuation of CPAP, surfactant to be given via thin catheter if FiO_2_ ≥ 0.30 while on CPAP of 7	Need for intubation and/any mechanical ventilation within 72 h	60 infants
Premie ProMISe [37] (NCT06007547)	22 0/7 to 29 6/7 weeks’ gestation, transitioned to CPAP support in the delivery room	Prophylactic surfactant 200 mg/kg within 15 min of birth via thin catheter	Continuation of CPAP, surfactant to be given via thin catheter if FiO_2_ ≥ 0.30 in first 48 h	Endotracheal intubation within 7 days	200 infants

*CPAP*: continuous positive airway pressure, *FEV*_1_: forced expiratory volume within 1 s.

## Directions for future studies on less invasive prophylactic surfactant

Future clinical trials evaluating less invasive prophylactic surfactant administration should prioritize enrolment of extremely preterm infants (24–27 weeks’ gestation) who exhibit stable heart rate and spontaneous breathing following effective initial resuscitation. Periviable infants (22–23 weeks’ gestation) typically face high rates of CPAP failure [38] attributable both to surfactant deficiency and to other physiological disturbances arising from extreme immaturity, including impaired respiratory mechanics and poor respiratory drive. As the care for these infants continues to improve and become the norm, future studies could expand to this group. As care for these infants is complex, these trials should be performed in centers which have significant experience with this cohort to ensure safety and feasibility. Conversely, more mature preterm infants born >=28 weeks have low CPAP failure rates [38] and low risk of developing BPD [39] making it unlikely that these infants would benefit from prophylactic surfactant. Numerous efficacy and safety outcomes should be evaluated in future trials of less invasive prophylactic surfactant. A selected list of proposed short- and long-term outcomes is outlined in Table 2.

**Table 2:** Selected clinical outcomes to be evaluated in future clinical trials on less invasive prophylactic surfactant.

Delivery room outcomes	Successful administration of prophylactic surfactant Adverse events during prophylactic surfactant administration Intubation in the delivery room
NICU outcomes	Death during first hospitalization Intubation in the first 72 h Mechanical ventilation during the first 72 h Receipt of repeat surfactant administration Total number of surfactant doses Method of surfactant administration Need for intubation at any time Duration of mechanical ventilation Duration of non-invasive respiratory support Duration of all forms of respiratory support Duration of oxygen supplementation Receipt of post-natal corticosteroids Development of BPD BPD grade Echocardiographically-proven pulmonary hypertension Tracheostomy placement Length of stay
Post-discharge outcomes	Oxygen therapy at home Respiratory disease in infancy Long term pulmonary function Neurodevelopment outcome

## Conclusion

Prophylactic surfactant administered less-invasively soon after birth offers a promising strategy to reduce respiratory morbidity in extremely preterm infants. Whether prophylactic surfactant via thin catheter should be routine for all extremely preterm infants or targeted to those at risk of CPAP failure requires further study. Additional research into aerosolized surfactant techniques and supraglottic airway devices for extremely preterm infants is also needed.
